# Occurrence of multidrug-resistant and ESBL-producing atypical enteropathogenic *Escherichia coli* in China

**DOI:** 10.1186/s13099-018-0234-0

**Published:** 2018-02-27

**Authors:** Yanmei Xu, Hui Sun, Xiangning Bai, Shanshan Fu, Ruyue Fan, Yanwen Xiong

**Affiliations:** 1 State Key Laboratory of Infectious Disease Prevention and Control, National Institute for Communicable Disease Control and Prevention, Chinese Center for Disease Control and Prevention, Changping, Beijing, China; 2 Collaborative Innovation Center for Diagnosis and Treatment of Infectious Diseases, Hangzhou, Zhejiang China

**Keywords:** Enteropathogenic *E. coli*, Antimicrobial resistance, Multidrug resistance, ESBL

## Abstract

**Background:**

Atypical enteropathogenic *Escherichia coli* (aEPEC) is regarded as a globally emerging enteropathogen. aEPECs exhibit various level of resistance to a range of antibiotics, which is increasing alarmingly. The present study investigated the antimicrobial resistance of aEPEC isolates recovered from diarrheal patients, healthy carriers, animals, and raw meats.

**Results:**

Among 267 aEPEC isolates, 146 (54.7%) were resistant to tetracycline, followed by ampicillin (49.4%), streptomycin (46.1%), and piperacillin (41.2%). Multidrug resistance (MDR) was detected in 128 (47.9%) isolates, and 40 MDR isolates were resistant to ≥ 10 antimicrobial agents. A total of 47 (17.6%) aEPEC isolates were identified as extended-spectrum β-lactamase (ESBL)-producers. The *bla*_CTX-M-14_ and *bla*_CTX-M-15_ genes were predominant among ESBL-producing isolates.

**Conclusions:**

This investigation depicted the occurrence of multidrug-resistant and ESBL-producing aEPEC isolates in China. The results suggested that it is necessary to continuously monitor the emergence and spread of MDR aEPEC.

**Electronic supplementary material:**

The online version of this article (10.1186/s13099-018-0234-0) contains supplementary material, which is available to authorized users.

## Background

*Escherichia coli* remains one of the most common etiological agents of diarrheal illness among children under 5 years old in developing countries [1, 2]. Six major diarrheagenic *E. coli* are well-characterized: enteropathogenic *E. coli* (EPEC), enterohemorrhagic *E. coli* (EHEC), enterotoxigenic *E. coli* (ETEC), enteroaggregative *E. coli* (EAEC), enteroinvasive *E. coli* (EIEC), and diffusely adherent *E. coli* (DAEC) [3]. EPEC are the primary cause of summer diarrhea in infants in developing countries [4]. It was estimated that about 79,000 deaths every year are linked with EPEC, which was the first to be identified and is the most prevalent pathotype of diarrheagenic *E. coli* [5].

EPEC isolates carry the locus of enterocyte effacement (LEE) island, which can induce the hallmark histopathology on the surfaces of intestinal epithelial cells, known as the attaching and effacing (A/E) lesion. A/E results in electrolyte disruption and eventual diarrhea [3, 6, 7]. Some EPEC isolates possess the adherence factor (EAF) plasmid, which carries the bundle-forming pilus genes, the plasmid-encoded regulator genes, and other virulence-related factors [3]. Depending on the presence or absence of the EAF plasmid, EPEC strains are divided into two subgroups: typical EPEC (tEPEC) and atypical EPEC (aEPEC) [8]. In developing countries, tEPEC was considered to be the main cause of infantile diarrhea for decades [6]. However, further studies have shown an apparent increase in the involvement of aEPEC strains in endemic childhood diarrhea and outbreaks in adults in recent years [9–14]. Thus, aEPEC strains have been regarded as emerging enteropathogens and have caused a number of infections [15–17]. Humans and animals, including food-production animals and pet animals, can be reservoirs of aEPEC, while the major reservoirs of tEPEC are humans [6].

Multidrug resistance (MDR), which was designated as resistance to one agent in three or more antibiotic classes [18], has been increasing alarmingly in *E. coli* (http://www.ecdc.europa.eu/en/healthtopics/antimicrobial_resistance/database/Pages/map_reports.aspx) [19]. The establishment of MDR is mediated by many diverse and interactive mechanisms, e.g., drug efflux, enzymatic inactivation, and target protection [20]. The determinants responsible for MDR are widely distributed among *E. coli* isolates, irrespective of their resources [20]. Production of extended-spectrum β-lactamase (ESBL) is one of the main mechanisms conferring the spread of MDR [21], because most ESBL-producing isolates show extensive resistance to other antimicrobial agents [22]. The genes encoding ESBLs are usually located on plasmids and different types of ESBLs have been identified globally [23]. According to their amino acid sequences, ESBLs are classified into several types, such as TEM, SHV, CTX, OXA, PER, and GES [24]. Currently, the most frequently detected genetic type of ESBL is CTX-M [25]. There are five major sublineages of CTX-M: 1, 2, 8, 9, and 25 [26].

The spread of antibiotic resistance among pathogens has become an emerging public health concern [21]. In China, aEPEC appeared to be one of the most common pathogens associated with infectious diarrhea [27]. However, there are few data available regarding the resistance of aEPEC. The present study aimed to determine the overall antimicrobial resistance profiles, the current prevalence of MDR, the ESBL genotype distribution, and the determinants of resistance in aEPEC isolates recovered from diarrheal patients, healthy carriers, animals, and raw meat in China. The results will fill in large knowledge gaps concerning this pathogen in China, and provide further information and guidance for the application antimicrobials in farm animals and in clinical treatment.

## Methods

### Isolation and identification of aEPEC isolates

Samples from different sources (diarrheal patients, healthy carriers, animals, and raw meat) were collected during 2006–2015 in ten geographical regions (Henan, Shanxi, Heilongjiang, Beijing, Qinghai, Guangdong, Sichuan, Shanghai, Guizhou, and Anhui) of China. Fecal samples of diarrheal patients were collected when patients were admitted to sentinel hospitals; stools from healthy carriers were sampled during routine physical examinations; while stool samples of animals and raw meat samples were collected during routine surveys.

The samples were processed as previously described [28]. In brief, the overnight enrichment culture of each sample was centrifuged and the cells were lysed in lysis buffer (10 mM Tris–HCl [pH 8.3], 100 mM NaCl, 1 mM EDTA [pH 9.0], 1% Triton X-100). The released DNA was then examined for *eae* gene by polymerase chain reaction (PCR) assays. The enrichment culture with *eae*^+^ were streaked on CHROMagar™ ECC plate (CHROMagar Co., Paris, France) and incubated at 37 °C for 18–24 h. Ten *E. coli*-like colonies from each culture were selected to detect the presence of the *eae* gene. The *eae*^+^ colonies were then subcultured on Luria–Bertani (LB) (Oxoid, Basingstoke, UK) plates, incubated for another 18–24 h, and subjected to PCR assays for the *eae*, *stx*_1_, *stx*_2_, and *bfpA* genes. Isolates that were *eae* positive, but *bfpA* and *stx*_1_/*stx*_2_ negative, were defined as aEPEC [6].

A total of 267 aEPEC isolates were identified and included in this study (Additional file 1). Among them, 151, 32, and 51 isolates were recovered from the stools of diarrheal patients, healthy carriers, and animals (cattle, pig, chicken, bird, pika, and marmot), respectively. The remaining 33 strains were isolated from raw meat (beef, pork, mutton, and chicken meat).

### Phenotypic antimicrobial susceptibility testing

Susceptibility to a panel of 23 drugs belonging to 12 classes was determined using the disc diffusion method in accordance with the Clinical and Laboratory Standards Institute (CLSI) (2017) [29]: penicillins: ampicillin (AM, 10 μg), piperacillin (PRL, 100 μg); β-lactam/β-lactamase inhibitor combinations: amoxicillin–clavulanic acid (AMC, 20/10 μg), ampicillin–sulbactam (SAM, 10/10 μg); cephems: cefepime (FEP, 30 μg), cefotaxime (CTX, 30 μg), ceftriaxone (CRO, 30 μg), cefuroxime (CXM, 30 μg), ceftazidime (CAZ, 30 μg); monobactams: aztreonam (ATM, 30 μg); carbapenems: imipenem (IPM, 10 μg), meropenem (MEM, 10 μg); aminoglycosides: gentamicin (CN, 10 μg), kanamycin (K, 30 μg), streptomycin (S, 10 μg); tetracyclines: tetracycline (TE, 30 μg); quinolones: nalidixic acid (NA, 30 μg); fluoroquinolones: ciprofloxacin (CIP, 5 μg), norfloxacin (NOR, 10 μg), levofloxacin (LEV, 5 μg); folate pathway inhibitors: trimethoprim–sulfamethoxazole (SXT, 1.25/23.75 μg); phenicols: chloramphenicol (C, 30 μg); and nitrofurans: nitrofurantoin (F, 300 μg) (Oxoid). *E. coli* ATCC^®^ 25922 served as the control. Strains were resuspended at a concentration of 0.5 McFarland standards in saline solution (0.85% NaCl) (BioMerieux, Marcyl’Etoile, France) and plated on Muller-Hinton agar plate (Thermo Fisher Scientific, Waltham, MA, USA) and grown at 37 °C for 16–18 h. Using a Scan 1200 (Interscience, Saint Nom, France), the diameters of the zone of inhibition were measured to the nearest 0.1 mm and recorded. Each isolate was determined as susceptible (S), intermediate (I), or resistant (R) according to the CLSI standards (2017). Isolates exhibiting resistance to at least one agent in three or more antimicrobial classes were defined as MDR strains [18].

### Screening and confirmation of ESBL producing isolates

ESBL production was screened phenotypically using cefotaxime (30 μg). The presumptive isolates were confirmed by combination disk tests with cefotaxime and ceftazidime (30 μg), with and without clavulanic acid (10 μg), as described by the CLSI guidelines [29]. A ≥ 5 mm increase in the zone diameter for cefotaxime or ceftazidime in combination with clavulanic acid versus the zone diameter of the corresponding antimicrobial agent alone defined an ESBL producer [29]. *Klebsiella pneumoniae* ATCC^®^ 700603 was used as a positive control.

### Identification of β-lactamase genes

DNA templates were prepared by crude extraction, as previously described [30]. All isolates were screened for the presence of the *bla*_CTX-M_ [26], *bla*_TEM_, and *bla*_SHV_ [31] gene using PCR. Four sets of group-specific primers were further used to identify five subgroups (*bla*_CTX-M-1_, *bla*_CTX-M-2_, *bla*_CTX-M-8/25/26_, and *bla*_CTX-M-9_) of *bla*_CTX-M_ [26]. The PCR products were resolved on a 1% agarose gel and then subjected to sequencing using an ABI 3730 Automated DNA Analyzer (Applied Biosystems, Foster City, CA, USA). The resulting sequences were compared against the sequences in GenBank (https://blast.ncbi.nlm.nih.gov/Blast.cgi).

### Whole genome sequencing and identification of antimicrobial resistance genes

Based on their serotypes, pulse-field gel electrophoresis (PFGE) patterns and multi-locus sequence typing (MLST), 96 isolates (69 from diarrheal patients, 16 from healthy carriers, and 11 from raw meat) were selected from among the 267 aEPEC strains for whole genome sequencing. Bacterial genomic DNA was extracted using a Wizard^®^ Genomic DNA Purification Kit (Promega Co., Madison, WI, USA) according to the manufacturer’s instructions. Genomic DNA was sequenced using an Illumina HiSeq 2500 PE125 instrument (Illumina, Santiago, CA, USA) with 500-bp libraries at the Beijing Novogene Bioinformatics Technology Co., Ltd. Coverage greater than 100× was obtained. The sequence read data was filtered by quality control using the Illumina data pipeline. High-quality filtered reads were assembled into contigs and scaffolds using SOAP de novo (http://soap.genomics.org.cn/soapdenovo.html). Based on the N90, N50, minimum contig size, maximum contig size, and number of contigs, the optimum genome assembly was chosen. Contigs with length > 500 bp were used for further analysis. Assembled draft genomes of all 96 isolates were then used to predict coding genes using the GeneMarkS program [32]. tRNAs and rRNAs were identified using tRNAscan-SE [33] and the rRNAmmer [34], respectively. Seven databases (Gene Ontology, Kyoto Encyclopedia of Genes and Genomes, Clusters of Orthologous Groups, Non-Redundant Protein Database, Transporter Classification Database, Swiss-Prot, and TrEMBL) were used to predict gene functions. The Antibiotic Resistance Genes Database (http://ardb.cbcb.umd.edu/) was used to search for antimicrobial resistance genes [35]. The raw data of these genomes have been submitted to the GenBank under accession numbers listed in Additional file 2.

### Statistical analysis

Differences in the antimicrobial resistance patterns among aEPEC origins were assessed by a two-tailed Chi square test or Fisher’s exact test, with a level of significance of *P* < 0.05. All statistical analyses were performed using Epi Info software, version 3.5.3 [36].

## Results

### Antimicrobial resistance of aEPEC isolates

Of the 267 aEPEC isolates tested, the highest levels of resistance were to tetracycline (54.7%), followed by ampicillin (49.4%), streptomycin (46.1%), and piperacillin (41.2%). Resistances against other antibiotics were as follows: trimethoprim–sulfamethoxazole (39.3%), nalidixic acid (35.2%), gentamicin (28.8%), kanamycin (14.6%), cefuroxime (19.5%), cefotaxime (18.4%), ceftriaxone (18.0%), and chloramphenicol (10.5%). However, most isolates were sensitive to cephalosporins (93.6% for cefepime and 97.0% for ceftazidime), fluoroquinolones (95.1% for ciprofloxacin, 96.6% for norfloxacin, and 95.5% for levofloxacin), and nitrofurantoin (98.5%). All isolates were susceptible to carbapenems (imipenem and meropenem) (Table 1, Additional file 1).

**Table 1:** Antimicrobial susceptibility profiles of 267 aEPEC strains isolated from different sources

Class/antimicrobial	No. of resistant isolates from different sources (%)				Total	*P* value
	Diarrheal patient (151)	Healthy carrier (32)	Animal (51)	Raw meat (33)		
Penicillins						
Ampicillin	84 (55.6)	15 (46.9)	20 (39.2)	13 (39.4)	132 (49.4)	0.1185
Piperacillin	71 (47.0)	13 (40.6)	19 (37.3)	7 (21.2)	110 (41.2)	0.0484
β-Lactam/β-lactamase inhibitor combinations						
Amoxicillin–clavulanic acid	14 (9.3)	0	10 (19.6)	3 (9.1)	27 (10.1)	0.0319
Ampicillin–sulbactam	24 (15.9)	0	11 (21.6)	9 (27.3)	44 (16.5)	0.0177
Cephems						
Cefepime	14 (9.3)	3 (9.4)	0	0	17 (6.4)	0.0396
Cefotaxime	39 (25.8)	5 (15.6)	1 (2.0)	4 (12.1)	49 (18.4)	0.0013
Ceftriaxone	38 (25.2)	5 (15.6)	2 (3.9)	3 (9.1)	48 (18.0)	0.0029
Ceftazidime	7 (4.6)	1 (3.1)	0	0	8 (3.0)	0.2622
Cefuroxime	39 (25.8)	5 (15.6)	2 (3.9)	6 (18.2)	52 (19.5)	0.0071
Monobactams						
Aztreonam	20 (12.6)	3 (9.4)	0	1 (3.0)	23 (8.6)	0.0202
Carbapenems						
Imipenem	0	0	0	0	0	–
Meropenem	0	0	0	0	0	–
Aminoglycosides						
Gentamicin	57 (37.7)	3 (9.4)	11 (21.6)	6 (18.2)	77 (28.8)	0.0019
Kanamycin	22 (14.6)	1 (3.1)	9 (17.6)	7 (21.2)	39 (14.6)	0.1782
Streptomycin	78 (51.7)	9 (28.1)	20 (39.2)	16 (48.5)	123 (46.1)	0.0692
Tetracyclines						
Tetracycline	89 (58.9)	15 (46.9)	21 (41.2)	21 (63.6)	146 (54.7)	0.0816
Quinolones						
Nalidixic acid	62 (41.1)	7 (21.9)	17 (33.3)	8 (24.2)	94 (35.2)	0.0866
Fluoroquinolones						
Ciprofloxacin	8 (5.3)	0	4 (7.8)	1 (3.0)	13 (4.9)	0.4053
Norfloxacin	5 (3.3)	0	4 (7.8)	0	9 (3.4)	0.1447
Levofloxacin	8 (5.3)	0	4 (7.8)	0	12 (4.5)	0.2020
Folate pathway inhibitors						
Trimethoprim–sulfamethoxazole	72 (47.7)	6 (18.8)	18 (35.3)	9 (27.3)	105 (39.3)	0.0060
Phenicols						
Chloramphenicol	11 (7.3)	0	9 (17.6)	8 (24.2)	28 (10.5)	0.0020
Nitrofurans						
Nitrofurantoin	3 (2.0)	0	1 (2.0)	0	4 (1.5)	0.7275

Although the isolates from different sources showed the highest resistance to tetracycline, the resistance rate of other antibiotics was different among isolates from diarrheal patients, healthy carriers, animals, and raw meat (Table 1). Of the 151 aEPEC strains isolated from diarrheal patients, 89 (58.9%) showed resistance to tetracycline, followed by ampicillin (55.6%), streptomycin (51.7%), trimethoprim–sulfamethoxazole (47.7%), piperacillin (47.0%), and nalidixic acid (41.1%).

Among the 32 strains isolated from healthy-carrier, resistances against tetracycline, ampicillin, and piperacillin were observed in 15 (46.9%), 15 (46.9%) and 13 (40.6%) isolates, respectively. In contrast, all isolates from healthy carriers were susceptible to β-lactam/β-lactamase inhibitor combinations (amoxicillin–clavulanic acid and ampicillin–sulbactam), fluoroquinolones (ciprofloxacin, norfloxacin and levofloxacin), chloramphenicol, and nitrofurantoin.

Of the 51 animal-originated strains, resistance to tetracycline was dominant (41.2%), followed by ampicillin (39.2%), streptomycin (39.2%), piperacillin (37.3%), and trimethoprim–sulfamethoxazole (35.3%). However, all 51 isolates were susceptible to cefepime, ceftazidime, and aztreonam.

Isolates from raw meat displayed the highest level of resistance to tetracycline (63.6%), followed by streptomycin (48.5%) and ampicillin (39.4%). However, all 33 isolates were susceptible to cefepime, ceftazidime, norfloxacin, levofloxacin, and nitrofurantoin.

### MDR aEPEC isolates

MDR was detected in 128 (47.9%) isolates. The prevalence of MDR was 55.6% (84/151), 31.3% (10/32), 37.3% (19/51), and 45.5% (15/33) among aEPEC isolates from diarrheal patients, healthy carriers, animals, and raw meat, respectively. Significant differences were observed in the overall distribution of MDR isolates among the four sources (χ^2^ = 9.563, *P* = 0.023). The prevalence of MDR in isolates from diarrheal patients was significantly higher than that from healthy carriers (χ^2^ = 6.282, *P* = 0.012) and animals (χ^2^ = 5.150, *P* = 0.023) (Table 2). Forty (31.3%) MDR isolates were resistant to ≥ 10 antimicrobial agents tested in the study. It was noteworthy that two patient isolates were resistant to 17 and 19 antibiotics, respectively.

**Table 2:** The distribution of multidrug resistance (MDR) strains among 267 aEPEC isolates

No. of antimicrobial group	No. of resistant isolates from different sources (%)				Total
	Diarrheal patient	Healthy carrier	Animal	Raw meat	
0	38 (25.2)	10 (31.3)	24 (47.1)	7 (21.2)	79 (29.6)
1–2	29 (19.2)	12 (37.5)	7 (13.7)	11 (33.3)	59 (22.1)
≥ 3	84 (55.6)	10 (31.3)	19 (37.3)	15 (45.5)	128 (47.9)
Total	151 (100)	32 (100)	51 (100)	33 (100)	267 (100)

### ESBL producing aEPEC isolates

**Fig. 1:**
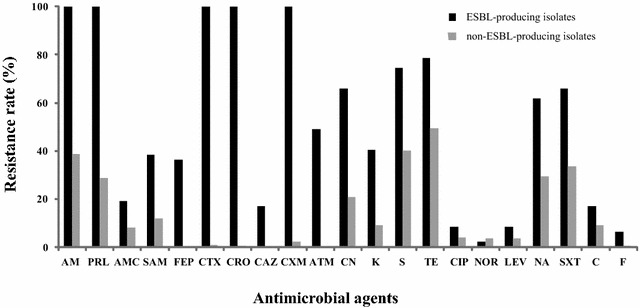
Comparison of antimicrobial susceptibility profiles of ESBL-producing and non-ESBL-producing aEPEC isolates. Proportions (%) of isolates (y-axis) resistant to antimicrobial agents (x-axis) among ESBL-producing and non-ESBL-producing isolates

A total of 47 (17.6%) ESBL-producing isolates were identified among 267 aEPEC isolates. The isolates from diarrheal patients showed the highest rate of ESBL-producing (38/151, 25.2%), compared to those from healthy carrier isolates (5/32, 15.6%), raw meat (3/33, 9.1%), and animals (1/51, 2.0%) (Table 3). Most (83.0%) ESBL-producing isolates were MDR strains. Compared with the non-ESBL producing isolates, ESBL producers displayed significantly higher rates of resistance to ampicillin, piperacillin, amoxicillin–clavulanic acid, ampicillin–sulbactam, cefepime, cefotaxime, ceftriaxone, ceftazidime, cefuroxime, aztreonam, gentamicin, kanamycin, streptomycin, tetracycline, nalidixic acid, trimethoprim–sulfamethoxazole, and nitrofurantoin (Fig. 1).

**Table 3:** Characteristics of 47 ESBL-producing aEPEC isolates

Origin (no. of isolates)	Isolates	Antimicrobial resistance pattern	*bla*_CTX-M_		*bla*_TEM_
			CTX-M-1 group	CTX-M-9 group	
Diarrheal patients (38)	EP004	AM, PRL, SAM, FEP, CTX, CRO, CXM, CAZ, ATM, CN, K, S, TE, NA, SXT	CTX-M-15	CTX-M-14	TEM-1
	EP008	AM, PRL, SAM, CTX, CRO, CXM, ATM, CN, K, S, TE, NA, SXT		CTX-M-14	TEM-1
	EP012	AM, PRL, SAM, CTX, CRO, CXM, ATM, CN, K, S, TE, NA, SXT		CTX-M-14	TEM-1
	EP013	AM, PRL, SAM, CTX, CRO, CXM, ATM, CN, K, S, TE, NA, SXT		CTX-M-14	TEM-1
	EP014	AM, PRL, SAM, CTX, CRO, CXM, ATM, CN, K, S, TE, NA, SXT		CTX-M-14	TEM-1
	EP017	AM, PRL, AMC, SAM, CTX, CRO, CXM, CN, K, S, TE, NA, SXT, C		CTX-M-14	TEM-1
	EP028	AM, PRL, FEP, CTX, CRO, CXM, CAZ, ATM, CN, TE, NA, SXT	CTX-M-15		TEM-1
	EP033	AM, PRL, CTX, CRO, CXM, CN, S, TE		CTX-M-14	TEM-1
	EP041	AM, PRL, CTX, CRO, CXM, CN, S, SXT	CTX-M-3		TEM-1
	EP043	AM, PRL, CTX, CRO, CXM, ATM	CTX-M-55		TEM-1
	EP064	AM, PRL, CTX, CRO, CXM, CN, NA, SXT	CTX-M-3		TEM-1
	EP074	AM, PRL, AMC, SAM, FEP, CTX, CRO, CXM, CAZ, ATM, CN, S, TE, NA, SXT	CTX-M-15		
	EP079	AM, PRL, AMC, SAM, FEP, CTX, CRO, CXM, CAZ, ATM, CN, K, S, TE, NA, SXT	CTX-M-15		TEM-1
	EP088	AM, PRL, FEP, CTX, CRO, CXM, CN, S, TE, SXT	CTX-M-3		
	EP103	AM, PRL, SAM, FEP, CTX, CRO, CXM, ATM, CN, K, S, TE, NA, CIP, LEV, C, F	CTX-M-55	CTX-M-14	TEM-1
	EP105	AM, PRL, CTX, CRO, CXM, ATM, CN, K, S, TE, NA, CIP, LEV, SXT, F		CTX-M-65	
	EP109	AM, PRL, AMC, CTX, CRO, CXM, ATM, CN, S, NA, SXT	CTX-M-15		TEM-1
	EP112	AM, PRL, SAM, CTX, CRO, CXM, CN, S, TE, NA, SXT		CTX-M-14	
	EP115	AM, PRL, AMC, SAM, FEP, CTX, CRO, CXM, ATM, CN, K, S, TE, NA, CIP, LEV, SXT, C, F	CTX-M-15		TEM-1
	EP116	AM, PRL, AMC, FEP, CTX, CRO, CXM, CN, S, TE, NA, SXT		CTX-M-14	TEM-1
	EP136	AM, PRL, CTX, CRO, CXM, ATM	CTX-M-15		
	EP155	AM, PRL, CTX, CRO, CXM, CN, S, TE, SXT		CTX-M-14	
	EP163	AM, PRL, CTX, CRO, CXM, S, SXT		CTX-M-14	
	EP166	AM, PRL, CTX, CRO, CXM, TE, SXT		CTX-M-14	TEM-1
	EP171	AM, PRL, FEP, CTX, CRO, CXM, CAZ, ATM, CN, TE, SXT	CTX-M-55		TEM-1
	EP176	AM, PRL, FEP, CTX, CRO, CXM, CN, K, S, TE, NA		CTX-M-14	TEM-1
	EP179	AM, PRL, SAM, CTX, CRO, CXM, CN, S, TE, NA, SXT		CTX-M-14	TEM-1
	EP180	AM, PRL, FEP, CTX, CRO, CXM, CAZ, ATM, S, TE, NA	CTX-M-15	CTX-M-14	
	EP182	AM, PRL, SAM, CTX, CRO, CXM, CN, K, S, TE, NA		CTX-M-14	
	EP186	AM, PRL, CTX, CRO, CXM, CN, K, S, TE, NA, CIP, NOR, LEV, SXT, C		CTX-M-14	TEM-1
	EP187	AM, PRL, AMC, FEP, CTX, CRO, CXM, CN, S, TE, SXT	CTX-M-3		TEM-1
	EP191	AM, PRL, SAM, CTX, CRO, CXM, CN, K, S, TE, SXT		CTX-M-14	TEM-1
	EP193	AM, PRL, SAM, FEP, CTX, CRO, CXM, CAZ, ATM, CN, K, S	CTX-M-55		TEM-1
	EP239	AM, PRL, AMC, SAM, CTX, CRO, CXM, CN, S, TE, NA, SXT		CTX-M-14	TEM-1
	EP370	AM, PRL, FEP, CTX, CRO, CXM, CN, TE, NA, SXT		CTX-M-14	
	EP408	AM, PRL, SAM, CTX, CRO, CXM, ATM, S, TE, NA, SXT		CTX-M-14	TEM-1
	EP410	AM, PRL, CTX, CRO, CXM, ATM, CN, NA	CTX-M-15		
	EP412	AM, PRL, CTX, CRO, CXM, TE		CTX-M-13	
Healthy carriers (5)	EP318	AM, PRL, FEP, CTX, CRO, CXM, CAZ, ATM	CTX-M-15		
	EP361	AM, PRL, FEP, CTX, CRO, CXM, K, S, TE, NA		CTX-M-14	
	EP 402	AM, PRL, FEP, CTX, CRO, CXM, ATM, TE	CTX-M-3		
	EP404	AM, PRL, CTX, CRO, CXM, S		CTX-M-14	
	EP415	AM, PRL, CTX, CRO, CXM, ATM, TE	CTX-M-15		
Animal (1)	EP298	AM, PRL, CTX, CRO, CXM, K, S, TE, NA, SXT, C		CTX-M-14	
Raw meat (3)	EP244	AM, PRL, AMC, SAM, CTX, CRO, CXM, ATM, S, TE, NA, SXT, C		CTX-M-14	
	EP299	AM, PRL, CTX, CRO, CXM, K, S, TE, NA, SXT, C		CTX-M-14	
	EP344	AM, PRL, CTX, CRO, CXM, K, S, TE, C		CTX-M-14

### Molecular characteristics of ESBL genes

The presence of *bla*_CTX-M_, *bla*_TEM_, and *bla*_SHV_ genes in 47 ESBL-producing isolates was screened using PCR. The *bla*_CTX-M-1_ subgroup was identified in 20 (42.6%) ESBL-producing isolates, with 17 from diarrheal patients and three from healthy carriers. The *bla*_CTX-M-9_ subgroup was found in 30 (63.8%) isolates, with 24 from diarrheal patients, three from raw meat, two from healthy carriers, and one from animals. A total of 26 isolates recovered from diarrheal patients possessed the *bla*_TEM_ subgroup (Table 3). None of the 47 isolates examined in this study was positive for the genes belonging to subgroups *bla*_CTX-M-2_, *bla*_CTX-M-8/25/26_, or *bla*_SHV_.

DNA sequencing showed that *bla*_CTX-M-14_ gene was the most prevalent, and was present in 28 (59.6%) ESBL-producing isolates, with 22 from diarrheal patients, three from raw meat, two from healthy carriers, and one from animal. The *bla*_CTX-M-15_ gene was identified in 11 (23.4%) isolates, with nine from diarrheal patients and two from healthy carriers. The *bla*_CTX-M-55_ and *bla*_CTX-M-3_ genes were found in four and five isolates, respectively. The two genes, *bla*_CTX-M-13_ and *bla*_CTX-M-65_, belonging to the subgroup *bla*_CTX-M-9_, were only found in two separate diarrheal patient-derived isolates. In addition, all of the 26 *bla*_TEM_ genes were identified as *bla*_TEM-1_. The coexistence of subgroup *bla*_CTX-M-1_ and *bla*_CTX-M-9_ genes was identified in three diarrheal patient isolates, including one that harbored *bla*_CTX-M-14_ and *bla*_CTX-M-55_, and two that harbored *bla*_CTX-M-14_ and *bla*_CTX-M-15_ (Table 3).

### Distribution of antimicrobial resistance determinants

Among the 96 genome-sequenced aEPEC isolates, 50 were resistant to ampicillin and possessed β-lactamase-related genes, including *bla*_TEM-1_ (48.0%), *bla*_CTX_ (16.0%), *bla*_OXA_ (6.0%), *bla*_TEM-1_ + *bla*_CTX_ (16.0%), *bla*_TEM-1_ + *bla*_LEN_ (2.0%), *bla*_CTX_ + *bla*_LEN_ (4.0%), and *bla*_CTX_ + *bla*_OXA_ (2.0%) (Table 4, Additional file 2). There was a significant association (χ^2^ = 84.715, *P* = 0.000) between the presence of these genes and resistance to ampicillin. Fifty-one isolates resistant to tetracycline harbored resistance associated determinants, including *tetA* (52.9%), *tetB* (3.9%), *tetC* (2.0%), *tetA* + *tetC* (17.6%), and *tetB* + *tetC* (10.0%). A significant association was observed between resistance to tetracycline and the occurrence of *tetA* (χ^2^ = 47.172, *P* = 0.000) and *tetB* (*P* = 0.062), but not with *tetC* (χ^2^ = 1.129, *P* = 0.288). Three and five chloramphenicol-resistant isolates harbored *cat* and *cml* genes, respectively. The *sul*1 + *dfra*12/17 (37.5%) and *sul*1 + *sul*2 + *dfra*5/12/17 (35.0%) were the predominant resistance genes among the 40 isolates that were resistant to trimethoprim–sulfamethoxazole. The combination of *sul* and *dfra* was detected more frequently in resistant strains than in sensitive strains (χ^2^ = 72.432, *P* = 0.000). The most frequent resistance gene observed in 33 phenotypically gentamicin-resistant isolates was *aac3iia* (69.7%). Four different genes or gene combinations, i.e., *ant3ia*, *aph33ib*, *aph33ib* + *aph6id*, and *aph33ib* + *aph6id* + *ant3ia*, were found in four (9.1%), two (4.5%), 24 (54.5%), and one (2.3%) of the 44 streptomycin-resistant isolates, respectively (Table 4, Additional file 2). Significant associations between the presence of these genes and streptomycin resistance were also observed (χ^2^ = 57.281, *P* = 0.000).

**Table 4:** Resistance-related genes among 96 genome sequenced aEPEC isolates

Phenotype of resistance (no. of isolates)	Resistance genes	No. of isolates (%)
Ampicillin (50)	*bla*_TEM-1_	24 (48.0)
	*bla*_CTX_	8 (16.0)
	*bla*_OXA_	3 (6.0)
	*bla*_TEM-1_ + *bla*_CTX_	8 (16.0)
	*bla*_TEM-1_ + *bla*_LEN_	1 (2.0)
	*bla*_CTX_ + *bla*_LEN_	2 (4.0)
	*bla*_CTX_ + *bla*_OXA_	1 (2.0)
Tetracycline (51)	*tetA*	27 (52.9)
	*tetB*	2 (3.9)
	*tetC*	1 (2.0)
	*tetA* + *tetC*	9 (17.6)
	*tetB* + *tetC*	5 (10.0)
Chloramphenicol (8)	*cat*	3 (37.5)
	*cml*	5 (62.5)
Trimethoprim–sulfamethoxazole (40)	*sul*1 + *dfra*12/17	15 (37.5)
	*sul*2 + *dfra*14/17	6 (15.0)
	*sul*3 + *dfra*12	3 (7.5)
	*sul*1 + *sul*2 + *dfra*5/12/17	14 (35.0)
	*dfra*1	2 (5.0)
	*dfra*17	1 (2.5)
Gentamicin (33)	*aac3iia*	23 (69.7)
	*aac3iia* + *ant2ia*	3 (9.1)
	*aac3iia* + *aph3ia*	2 (6.1)
	*aac3iia* + *ant2ia* + *aph3ia*	1 (3.0)
Streptomycin (44)	*ant3ia*	4 (9.1)
	*aph33ib*	2 (4.5)
	*aph33ib* + *aph6id*	24 (54.5)
	*aph33ib* + *aph6id* + *ant3ia*	1 (2.3)
Kanamycin (12)	*ant2ia*	1 (8.3)
	*aph3ia*	3 (25.0)
	*ant2ia* + *aph3ia*	2 (16.7)

## Discussion

Globally, EPECs displaying different levels of resistance to a range of antibiotics are increasing alarmingly [37]. The antimicrobial resistance of EPEC has been reported in many countries, including Brazil [38, 39], India [40], Iran [41], Ireland [42], the United Kingdom [43], and Singapore [44]. In China, only two studies are available: one characterizing 39 EPEC isolates in ready-to-eat foods [45] and another examining 58 EPEC isolates recovered from pediatric diarrheal patients [46]. These EPEC strains were either restricted to being from foods or were regionally restricted. In the present study, the 267 aEPEC isolates were recovered from different sources (diarrheal patients, healthy carriers, animals and raw meat) from ten provinces/cities of China. This was the first study to reveal the comprehensive antimicrobial resistance of aEPEC in China and to provide further insight into the current situation of this specific diarrheagenic *E. coli*.

Of the 151 diarrheal patient-derived aEPEC isolates, the highest resistance rate was to tetracycline, followed by ampicillin and streptomycin, which was different from reports in Iran [47], Brazil [39], and India [40]. Physicians in China should pay attention to the antimicrobial resistance of clinical aEPEC isolates, because EPEC is still one of the most common pathogens associated with infectious diarrhea. Domestic animals, such as sheep, cattle, poultry, and pigs, have been considered as the main reservoirs of aEPEC [14]. In Europe, the predominant antimicrobial agents administered to animals are sulphonamides and/or trimethoprim, tetracyclines and β-lactams [48]. However, there is little antimicrobials consumption data available in this field in China. It was reported that high doses and multiple types of veterinary antimicrobial products were used routinely in livestock husbandry [49]. The agents mentioned above are also included in the antimicrobials that can be used in the treatment and prevention of animal diseases. The high prevalence of antimicrobial-resistant aEPEC in raw meat and animals could be explained by the possible overuse and misuse of tetracyclines, ampicillin, and trimethoprim/sulphonamides in veterinary practice and agriculture. Poor sanitary conditions or practices might also play a role in the spread of resistant aEPEC.

The emergence of multidrug resistance, especially among *Enterobacteriaceae*, i.e., *E. coli*, has become a critical public concern [18]. In this study, nearly half of the 267 aEPEC strains were multidrug resistant. These MDR strains showed high resistance to tetracycline (92.2%) and ampicillin (89.8%), and 31.3% of that showed resistance to ≥ 10 antimicrobial agents. In addition, in this study, significantly more aEPEC strains from diarrheal patients showed multidrug resistance than did strains from healthy carriers and animals. Thus, diarrheal patients may be the main source of MDR aEPEC strains in China and clinicians should be careful when using antibiotics as therapy for EPEC infections. A recent study showed that wild birds could also act as carriers of MDR EPEC [50]. Consistent with this, we found that 19 (37.3%) aEPEC strains from animals, including birds, pika, and marmot, were MDR. In this sense, MDR aEPEC could emerge in the natural environment and then pose potential risk to public health.

Most multidrug resistances in *Enterobacteriaceae* are associated with ESBLs [51]. *E. coli* has become one of main producers of ESBL and has posed a major challenge in the treatment of bacterial infection [19]. A previous study showed that occurrence of ESBL-producing *E. coli* in patients in China varied from 30.2 to 57.0% [52]. In our study, 47 (17.6%) aEPEC isolates were identified as ESBL-producing strains, with 38 the isolates coming from diarrheal patients. Most ESBL-producing isolates showed co-resistance to other antimicrobial agents, such as aminoglycosides, tetracyclines, and sulfonamides, and even to fluoroquinolones [22]. The present results showed that ESBL-producing aEPEC isolates displayed co-resistance to aminoglycosides, tetracycline, nalidixic acid, trimethoprim–sulfamethoxazole, and nitrofurantoin, but not to fluoroquinolones. It is worth noting that MDR *E. coli* usually implies significant increase of resistance and pathogenic potential, such as the emergence of ESBL-producing clone ST131 [53] and another clinically relevant ESBL-producing clone ST410 [54]. The multi-locus sequence typing (MLST) analysis in our previous study indicated that these aEPEC isolates showed high clonal diversity, but none of them were identified as ST131 or ST410 [28].

TEM, SHV, and CTX-M are the three main genetic types of ESBLs [19]. Currently, the CTX-M-type ESBLs have dramatically increased and largely outnumber other types of ESBLs [25]. However, there are extensive geographical variations in the distribution of dominant CTX-M types across different countries, such as CTX-M-2 in Japan, CTX-M-1 in Italy, and CTX-M-2 and CTX-M-15 in Brazil. By contrast, CTX-M-15 widespread throughout the world [22, 55, 56]. In the present study, all 47 ESBL-producing aEPEC isolates possessed CTX-M genes. No TEM or SHV type ESBL genes were detected. The most prevalent gene was *bla*_CTX-M-14_, followed by *bla*_CTX-M-15_, with majority being from diarrheal patients. These findings revealed that CTX-M-14 and CTX-M-15 were predominant among aEPEC isolates in China. This is consistent with previous reports that CTX-M-14 was the most abundant CTX-M type among *E. coli* strains from animals [57] and clinical patients in China [52]. CTX-M-55 was observed only in four aEPEC strains from diarrheal patients, although it was demonstrated to be widespread in *E. coli* isolates from food-producing animals and environmental samples in China [58, 59]. These findings suggested that humans might acquire these strains from animals, as well as from the food chain.

High levels of resistance to tetracycline, ampicillin, and streptomycin were identified among 96 genome sequenced aEPEC isolates. More than half of the ampicillin resistant strains harbored the *bla*_TEM-1_ gene in this study. It has been reported that *bla*_TEM_ was the most frequent β-lactamase gene involved in ampicillin resistance in *E. coli* [60]. Of the known tetracycline resistance genes, only *tetA*, *tetB*, and *tetC* (alone or in combination) were detected, indicating that the major mechanism involved in tetracycline resistance in aEPEC isolates is active efflux. This is consistent with the investigation of EPEC from diarrheic rabbits in Portugal [60]. Among the aEPEC resistant to aminoglycosides, 69.7% of the isolates resistant to gentamicin carried *aac3iia*; 54.5% isolates resistant to streptomycin possessed genes *aph33ib* and *aph6id*; and most isolates resistant to kanamycin harbored *aph3ia*. These results suggested that aminoglycoside acetyltransferases are the main mechanism of resistance to gentamicin, while aminoglycoside phosphotransferases are the predominant mechanism mediating streptomycin and kanamycin resistance. With respect to determinants responsible for resistance to trimethoprim–sulfamethoxazole, our results demonstrated that *sul*1, *sul*2, *dfra*12, and/or *dfra*17 were the predominant genes, as revealed by a previous study [60].

Some limitations exist in this study. Compared with the number of strains from diarrheal patients, fewer isolates from healthy carriers, animals, and raw meat were included. Further investigations are needed to clarify the association between virulence and antimicrobial resistance.

In conclusion, our investigation revealed the occurrence of multidrug-resistant and ESBL-producing aEPEC isolates in China. These results suggest that it is necessary to continuously monitor the emergence and spread of MDR aEPEC to guide the application of antimicrobials in farm animals and in clinical treatment.

## Additional files

**Additional file 1.** Antimicrobial susceptibility of 267 aEPEC strains tested in the study.

**Additional file 2.** Antimicrobial susceptibility profiles and resistance-related genes of 96 genome-sequenced aEPEC strains.

## Abbreviations

A/E: attaching and effacing
AM: ampicillin
AMC: amoxicillin–clavulanic acid
ATM: aztreonam
aEPEC: atypical EPEC
C: chloramphenicol
F: nitrofurantoin
CAZ: ceftazidime
CIP: ciprofloxacin
CN: gentamicin
CRO: ceftriaxone
CTX: cefotaxime
CXM: cefuroxime
EPEC: enteropathogenic *Escherichia coli*
ESBL: extended-spectrum β-lactamase
FEP: cefepime
K: kanamycin
LEE: locus of enterocyte effacement
LEV: levofloxacin
MDR: multidrug resistance
MEM: meropenem
NA: nalidixic acid
NOR: norfloxacin
IPM: imipenem
PRL: piperacillin
S: streptomycin
SAM: ampicillin–sulbactam
SXT: trimethoprim–sulfamethoxazole
TE: tetracycline
tEPEC: typical EPEC
